# *Codonopsis pilosula* seedling drought- responsive key genes and pathways revealed by comparative transcriptome

**DOI:** 10.3389/fpls.2024.1454569

**Published:** 2024-10-30

**Authors:** Hongyan Wang, Yuan Chen, Lanlan Liu, Fengxia Guo, Wei Liang, Linlin Dong, Pengbin Dong, Jiali Cheng, Yongzhong Chen

**Affiliations:** ^1^ College of Agronomy, College of Life Science and Technology, State Key Laboratory of Aridland Crop Science, Gansu Agricultural University, Lanzhou, China; ^2^ College of Forestry Engineering, Guangxi Eco-engineering Vocational and Technical College, Nanning, China; ^3^ Institute of Chinese Materia Medica, China Academy of Chinese Medical Sciences, Beijing, China

**Keywords:** *Codonopsis pilosula*, drought stress, differentially expressed genes, comparative transcriptome, qRT-PCR

## Abstract

**Background:**

*Codonopsis pilosula* (Campanulaceae) is a traditional herbal plant that is widely used in China, and the drought stress during the seedling stage directly affects the quality, ultimately impacting its yield. However, the molecular mechanisms underlying the drought resistance of *C*. *pilosula* seedlings remain unclear.

**Method:**

Herein, we conducted extensive comparative transcriptome and physiological studies on two distinct *C. pilosula* cultivar (G1 and W1) seedlings subjected to a 4-day drought treatment.

**Results:**

Our findings revealed that cultivar G1 exhibited enhanced retention of proline and chlorophyll, alongside a marked elevation in peroxidase activity, coupled with diminished levels of malondialdehyde and reduced leaf relative electrolyte leakage compared with cultivar W1. This suggested that cultivar G1 had relatively higher protective enzyme activity and ROS quenching capacity. We discerned a total of 21,535 expressed genes and identified 4,192 differentially expressed genes (DEGs) by RNA sequencing (RNA-seq). Our analysis revealed that 1,764 DEGs unique to G1 underwent thorough annotation and functional categorization utilizing diverse databases. Under drought conditions, the DEGs in G1 were predominantly linked to starch and sucrose metabolic pathways, plant hormone signaling, and glutathione metabolism. Notably, the drought-responsive genes in G1 were heavily implicated in hormonal modulation, such as ABA receptor3-like gene (*PYL9*), regulation by transcription factors (*KAN4*, *BHLH80*, *ERF1B*), and orchestration of drought-responsive gene expression. These results suggest that cultivar G1 possesses stronger stress tolerance and can better adapt to drought growing conditions. The congruence between qRT-PCR validation and RNA-seq data for 15 DEGs further substantiated our findings.

**Conclusion:**

Our research provides novel insights into the physiological adaptations of *C. pilosula* to arid conditions and lays the groundwork for the development of new, drought-tolerant *C. pilosula* cultivars.

## 1 Introduction

The utilization of medicinal plants represents a significant approach for humans to harness natural resources. These plants not only serve as treatments for various diseases but also play a crucial role in boosting the immune system and preventing illness. As the global population grows and living standards improve, the demand for medicinal plants continues to rise. Notably, approximately 75% of the population in developing countries primarily relies on herbal medicines for their healthcare needs (Kim et al., 2015). *Codonopsis pilosula* is a traditional herbal plant that is widely used in Asian countries, mainly planted in China, Japan, and Korea (Chen and Huang, 2018; Liu et al., 2018), and its dry roots are used as medicine (*Codonopsis Radix*, also Dangshen) (C. P. Committee, 2020). Furthermore, the Chinese Pharmacopeia lists more than 110 Chinese Herbal Medicines (CHM) preparations containing *Codonopsis Radix* or its extracts (C. P. Committee, 2020) due to its extensive pharmacological effects, such as strengthening the spleen, benefiting the lungs, nourishing the blood, and promoting the production of body fluids (C. P. Committee, 2020; Gao et al., 2018; He et al., 2014; Sun et al., 2018a). People in countries such as China, Japan, North Korea, South Korea, and the United States also use it as a food additive in wine, soup, porridge, etc (Zou et al., 2014; Boo et al., 2017; Gao et al., 2018; Moon et al., 2018).

*C*. *pilosula* is a perennial herbaceous plant in the Campanulaceae family that propagates by seed reproduction (Shin et al., 2019) and is mainly cultivated in Gansu Province, China, which has an arid and semiarid temperate continental climate (He et al., 2013). Drought is one of the most important environmental stressors, and its intensity increases due to climate changes and irrigation water shortages caused by world population growth (Marthandan et al., 2020). The agricultural losses caused by drought each year account for approximately 70% of the global potential yield losses (Min et al., 2016; Wu et al., 2017), mainly due to water deprivation causing a decrease in water potential, nutrient uptake, and photosynthesis, and inducing oxidative damage from reactive oxygen species (ROS) and disturbance of metabolism, finally resulting in reduced production and yield quality of crops (Wahab et al., 2022). To cope with drought stress, plants initiate multiple drought- response strategies at the morphological, physiological, and molecular levels. These strategies include altering the structural characteristics of roots and leaves, enhancing the synthesis of hormones and osmotic regulators, and regulating the expression of drought-tolerant genes (Chimungu et al., 2014). The yield and quality of *Codonopsis Radix* depend on the *C. pilosula* seed and seedling quality. High-quality *Codonopsis* seedlings are fundamental for improving the *Codonopsis Radix* yield and quality (Liu et al., 2019; Xiao et al., 2020). The *C. pilosula* seedling stage requires more water than the medicinal value formative period, while drought stress at the seedling stage directly affects the quality of the *C*. *pilosula* and, ultimately, its yield. Thus, the needs of daily life and clinical medicine for this species cannot be met (He et al., 2015).

After years of field breeding, our research group has successfully cultivated a new cultivar of *C*. *pilosula*, ‘Gandang number 1, G1’. Experiments have shown that cultivar G1 has strong stress resistance, but the yield of its dried roots is not the highest (Wang et al., 2024). This clearly cannot meet the demand for high-yield and high-stress resistance new cultivars of *C*. *pilosula* in production. At present, assisted breeding based on biochemical markers is a valid approach used to accelerate the cultivation of *C*. *pilosula* cultivars (Gupta and Crants, 2019). Therefore, it is essential to elucidate the potential mechanisms underlying the drought stress response, as well as to identify the drought resistance genes unique to cultivar G1. This knowledge can then be applied in molecular marker-assisted breeding to develop new cultivars of *C. pilosula* that exhibit both high yield and enhanced resistance to stress.

In this work, we employed an Illumina RNA sequencing and analysis-based methodology to conduct a comprehensive comparative transcriptome analysis of two contrasting *C. pilosula* cultivars—the new cultivar G1 and the widely promoted local variety Weidang No. 1 (W1), both at the seedling stage. Our aim was to reveal the dynamic molecular mechanisms that underlie the drought stress responses in *C. pilosula* and to identify the drought resistance genes that are unique to the G1 cultivar. Additionally, we assessed physiological indices to provide a foundational reference for our research. Ultimately, this study offers a molecular basis for breeding new drought-resistant cultivars of *C. pilosula.*

## 2 Materials and methods

### 2.1 Plant materials and drought stress treatment

In this experiment, potted soil-cultivated *Codonopsis* seedlings were utilized. The soil used for the test, with a moisture content of 150.43 g/kg, was taken from the Bacchus Garden of Gansu Agricultural University. This loess soil exhibited an electrical conductivity of 173.67 μs/cm and a pH level of 8.19 and was enriched with 12.27 g/kg of organic matter. Additionally, it contained 17.08 mg/kg of nitrate nitrogen, 9.79 mg/kg of ammonium nitrogen, 13.48 mg/kg of available phosphorus, and 108.06 mg/kg of available potassium. The test cultivars of *C*. *pilosula* included Weidang No. 1 (W1), a variety promoted in Dingxi, Gansu Province (the seed was obtained from the Dry Farming Research and Promotion Center of Dingxi, Gansu Province), and Gandang No. 1 (G1), a cultivar selected by the group of Yuan Chen and Feng-xia Guo of Gansu Agricultural University. All seeds were naturally shade-dried seeds harvested in the same year. Seeds weighing 0.0430 ± 0.0005 g—sufficient for approximately 150 seeds—were sown in plastic pots. These pots, with dimensions of 13 cm in diameter, 12 cm in height, and a bottom diameter of 9 cm, were used for 20 pots per cultivar. A rain-proof shed was used to prevent rainwater from entering and to ensure consistency with the local climatic conditions.

During the potted plant experiment, the climate conditions were obtained from the European Centre for Medium Range Weather Forecasts (ECMWF, https://www.ecmwf.int/). The following data were collected: daily maximum temperature (°C), daily minimum temperature (°C), average humidity (% RH), average daily rainfall (mm), daily maximum Direct Normal Irradiance (DNI, W/m^2^), and daily minimum DNI (W/m^2^) data at the coordinates of the potted plant experiment (36.092035° N, 103.699152° E). The daily maximum solar intensity and daily minimum solar intensity data using formula 1 DNI (W/m^2^) = 126.58 solar intensity (lux). The soil moisture content was maintained by weighing during the experimental stage (Ruiz-Nieto et al., 2015). To minimize the effects of environmental heterogeneity, all pots were periodically moved and rotated (Ruiz-Nieto et al., 2015). The climatic conditions throughout the entire experimental period are summarized in **Supplementary Figure S1**.

The weighing method was employed to maintain the soil moisture content of potted plants at 180 ± 5 g/kg. Four weeks after the emergence of seeds, two pots of each cultivar were left as control for observation and photography. The remaining 18 pots of each cultivar underwent drought treatment (D) by withholding water, with the day water was withheld designated as drought day 0. Sampling was conducted at 4–5 pm on the same day. After sampling, the soil moisture content of the potted soil was immediately measured by the drying method. Three samples, with each pot containing one biological replicate, were collected on drought days 0, 2, 4, 6, and 7. The samples were immediately stored at −80°C for subsequent physiological analyses. Based on phenotype observations and physiological indicator measurement results, samples from drought days 0 (CG1, CW1) and 4 (DG1, DW1) were used for transcriptome sequencing and quantitative real-time PCR (qRT-PCR) assays.

### 2.2 Physiological and phenotypic characterizations

Soil moisture content was determined using the oven-drying method: Fresh soil samples were placed in an aluminum box of two-thirds’ capacity, weighed, and then oven dried at 105°C for 6 to 8 h, and the samples were reweighed after cooling. The formula was as follows: Soil moisture content (g/kg) = [(Weight of box + wet soil) −(Weight of box + dry soil)]/ [[(Weight of box + dry soil) − (Weight of box)] ×10].

Lipid peroxidation was measured following treatment and after 5 h by using malondialdehyde (MDA) equivalents (Dhindsa and Matowe, 1981). Leaves (0.20 g) from each treatment were ground in a precooled mortar with 10 mL of 0.05 M phosphate buffer (pH 7.0) and liquid nitrogen. The homogenate was evenly divided into two 5- mL portions using a standard pipette, and placed into separate 5-mL centrifuge tubes. One portion was immediately centrifuged at 12,000 ×g for 10 min to measure the initial MDA content. The other portion was incubated at room temperature with gentle shaking for 5 h before centrifugation and MDA measurement. For MDA quantification, the supernatant (1.5 mL) after centrifugation was mixed with an equal volume of a solution containing 20% trichloroacetic acid (TCA) and 0.6% thiobarbituric acid (TBA) in 0.05 M phosphate buffer (pH 7.0). The mixture was heated in a 95°C water bath for 15 min, and then rapidly cooled and centrifuged at 12,000 ×g for 10 min. The absorbance was measured at 450 nm, 532 nm, and 600 nm. The automatic oxidation rate (AR, = (5 h MDA-0 h MDA)/5) of membrane lipids was calculated as the average increase in the MDA concentration per hour (Li and Mei, 1989).

The levels of proline were evaluated using a Proline Assay Kit (A107-1-1, Jiancheng, Nanjing, China) by the colorimetric method. Ethanol extraction was used for chlorophyll determination, and the leaf relative electrolyte conductivity was determined using a P902 conductivity meter (Youke, Shanghai, China). Antioxidant enzymatic activity was performed at 4°C. The samples were homogenized in liquid nitrogen with 5 mL extract buffer containing 0.2 mM ethylenediaminetetraacetic (EDTA) and 2% polyvinylpyrrolidone (PVP) in 25 mM PBS buffer (pH 7.8). The homogenate was centrifuged at 15,000 r·min^−1^ for 20 min. The supernatant was used for antioxidant enzyme analysis (Guo et al., 2006, 2021; Niu et al., 2018). The activity for SOD was expressed as U/g, the activity for APX was expressed as U/s/g, and CAT and POD activities were expressed as U/min/g according to Niu et al. (2018).

### 2.3 RNA isolation, transcriptome sequencing, and data analysis

Total RNA was extracted from the leaf tissues by using TRIzol reagent following the manufacturer’s procedure (Invitrogen, CA, USA). RNA quality was evaluated on 1% agarose gel, and RNA concentrations were determined with NanoDrop 2000 spectrophotometer (Thermo Technologies) (Wei et al., 2019). High-quality RNA (RNA integrity number [RIN] scores >7.5) was used for subsequent experiments. The messenger RNA (mRNA) was enriched using Oligo (dT) beads, followed by fragmentation into short fragments using fragmentation buffer and reverse transcribed into cDNA with random primers. Second-strand cDNA was synthesized by DNA polymerase I, RNase H, dNTP, and buffer. Subsequently, the cDNA fragments were purified with the Qiaquick PCR extraction kit (Qiagen, Venlo, Netherlands), end repaired, A base added, and ligated to Illumina sequencing adapters. The ligation products were size selected (~300 bp) by agarose gel electrophoresis, PCR amplified, and sequenced using Illumina NovaSeq 6000. Raw reads were processed by quality control with unjoining and quality shearing to obtain the clean reads. The clean reads of each sample were assembled using Trinity v2.5.0 to acquire the number of unigenes. All the assembled transcripts were annotated against the following databases: NCBI_NR (non-redundant protein sequences), Gene Ontology (GO), and KEGG (Kyoto Encyclopedia of Genes and Genomes). The expression of unigenes was summarized using RSEM1.2.31. Significantly differentially expressed genes (DEGs) were analyzed using the DESeq2 1.10.1 R package. DEGs were identified within the following criteria: adjusted P-value < 0.005, |log2(foldchange)| ≥ 2 (Robinson et al., 2010). The acquired DEGs then underwent KEGG enrichment analysis using KOBAS (http://kobas.cbi.pku.edu.cn/kobas3) to identify the biological functions and related metabolic pathways. The raw transcriptome data have been deposited in the NCBI Gene Expression Omnibus (GEO) under the accession number GSE270657.

### 2.4 Quantitative real-time PCR analysis

To validate the reliability of the RNA-Seq transcriptome sequencing results from our experiments, we randomly selected 15 genes and quantified their expression levels using quantitative real-time fluorescence PCR (qRT-PCR). Primer design was conducted using Primer 5.0 software (**Supplementary Table S1**), with 18S rRNA serving as the internal reference gene. RNA from each sample group was reverse transcribed using a reverse transcription kit from TaKaRa. The resulting cDNA was subsequently stored at −20°C. For qRT-PCR reactions, qPCR Mix 15 µL, 2 μL Mg^2+^ (25 mM), 0.5 µL each for forward and reverse primers (10 µM), cDNA 2 µL, and dd H_2_O 10.0 µL were included, and the PCR amplification program is shown in **Supplementary Table S2**. The relative expression 2^−ΔΔCt^ levels were calculated using the relative method (Livak and Schmittgen, 2001).

### 2.5 Statistical analysis of physiological data

We used the SPSS software package (version 22.0; SPSS Institute Ltd., Armonk, NY, USA) to conduct the statistical analysis of physiological data by Fisher’s protected least significant differences (PLSD) test. The level of significance was set at *P* ≤ 0.05.

## 3 Results

### 3.1 Phenotypical response of two contrasting *C. pilosula* cultivars to drought stress

**Figure 1:**
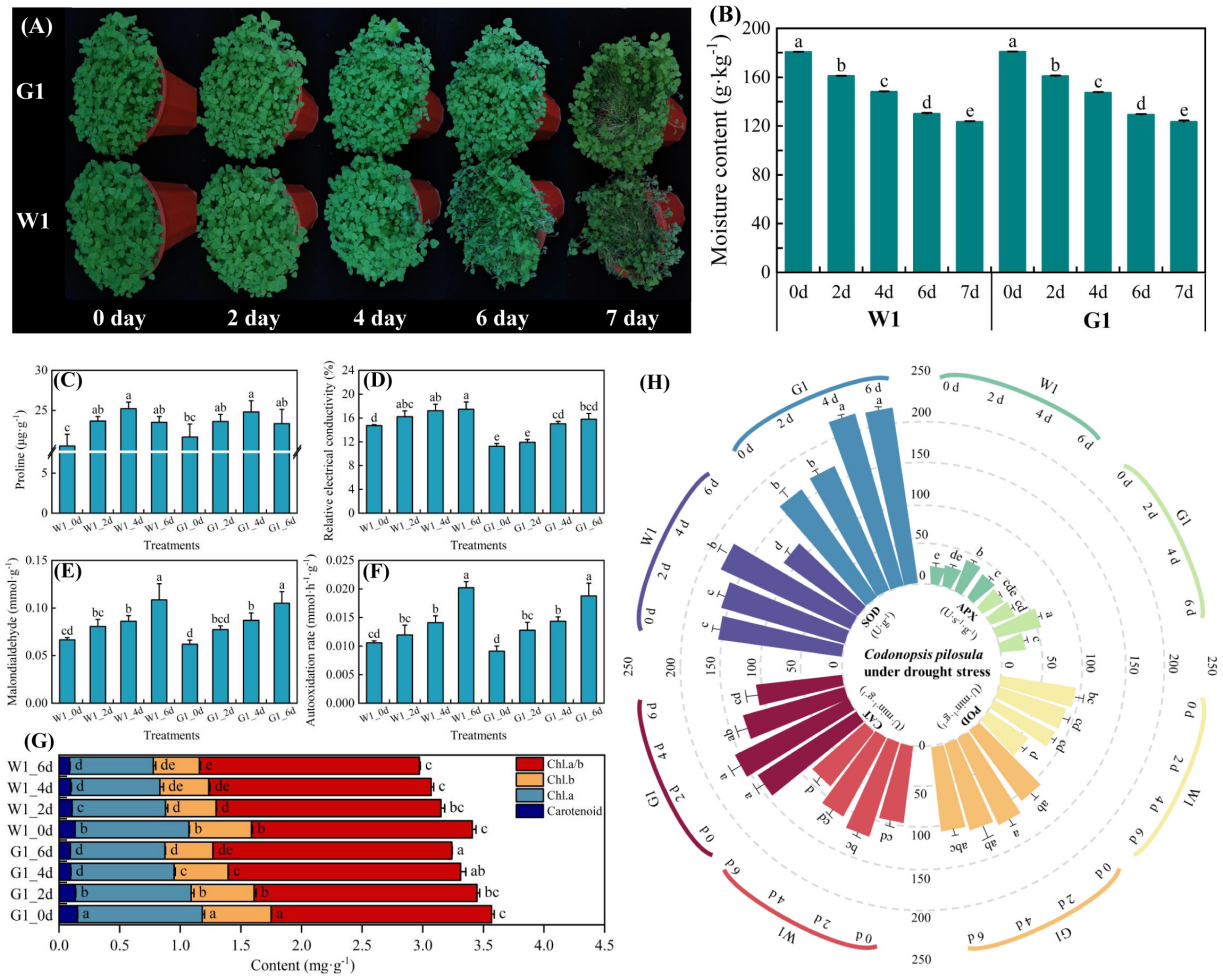
Phenotypical characterization of two cultivars under drought stress. **(A)** Phenotypes of W1 and G1 on days 0, 2, 4, 6 and 7 of drought treatment. **(B)** Soil moisture content on days 0, 2, 4, 6, and 7 of the drought treatment. **(C–F)** Effect on osmotic adjustment substances of proline, relative electrical conductivity, malondialdehyde (MDA), and automatic oxidation rate. **(G)** Changes in chlorophyll. **(H)** Antioxidant enzyme activity of peroxidase (POD), catalase (CAT), ascorbate peroxidase (APX), and superoxide dismutase (SOD). Data are mean ± SD (n = 3). Different lowercase letters mean a significant difference at *P*< 0.05.

To investigate the drought tolerance phenotype of *C*. *pilosula* cultivars W1 and G1, 4-week-old seedlings were subjected to drought treatment. After 6 days of drought exposure, the leaves and stems of cultivar W1 exhibited significant wilting, with the stems showing more severe dehydration than the leaves. In contrast, the leaves and stems of cultivar G1 remained in relatively good condition (**Figure 1A**). Following 7 days of drought treatment, the stems of cultivar W1 were nearly completely dehydrated, whereas the leaves and stems of cultivar G1 began to show initial signs of wilting.

In both *C*. *pilosula* cultivars, proline content and antioxidant enzyme activities initially increased from days 1 to 4, followed by a decrease on day 6 as the duration of stress exposure lengthened (**Figures 1C, H**). The chlorophyll content peaked on day 2 of drought stress before subsequently declining (**Figure 1G**). Additionally, other leaf osmotic adjustment substances, including relative electrical conductivity, malondialdehyde, and automatic rate, exhibited a significant increase (*P* < 0.05) during the drought period from days 1 to 6 (**Figures 1D–F**). In comparison, cultivar G1 consistently maintained higher levels of proline content, antioxidant enzyme activities, and chlorophyll content than cultivar W1 at all time points. However, the levels of other osmotic adjustment substances were lower in cultivar G1 than in cultivar W1. This observation suggests that as the duration of drought exposure increases, the leaf cell membranes of cultivar W1 may experience significant damage, leading to the release of cell membrane lipids and the destruction of membrane structure (Zenda et al., 2018). Therefore, after evaluating the physiological changes, we conducted transcriptome analysis on leaf samples of two *C*. *pilosula* cultivars, comparing those with normal growth (control, C) and those subjected to drought stress on day 4 (D). This analysis aimed to identify the relevant genes associated with drought stress in *C. pilosula* seedlings.

### 3.2 Transcriptomic analysis of *C*. *pilosula* response to drought stress

Transcriptomic analysis was used to explore the molecular mechanisms underlying the adaptation of *C. pilosula* Seedlings to drought stress. In total, 549.75 million raw sequencing reads were generated. After removing adapter sequences and low-quality reads, we obtained 71.44 GB of clean data; all Q20 values and Q30 values were more than 98% and 94% (**Supplementary Table S3**). Approximately 55,270 genes were assembled, with lengths ranging from 201 bp to 9,071 bp. All of these genes were successfully annotated in the database (**Supplementary Table S4**).

**Figure 2:**
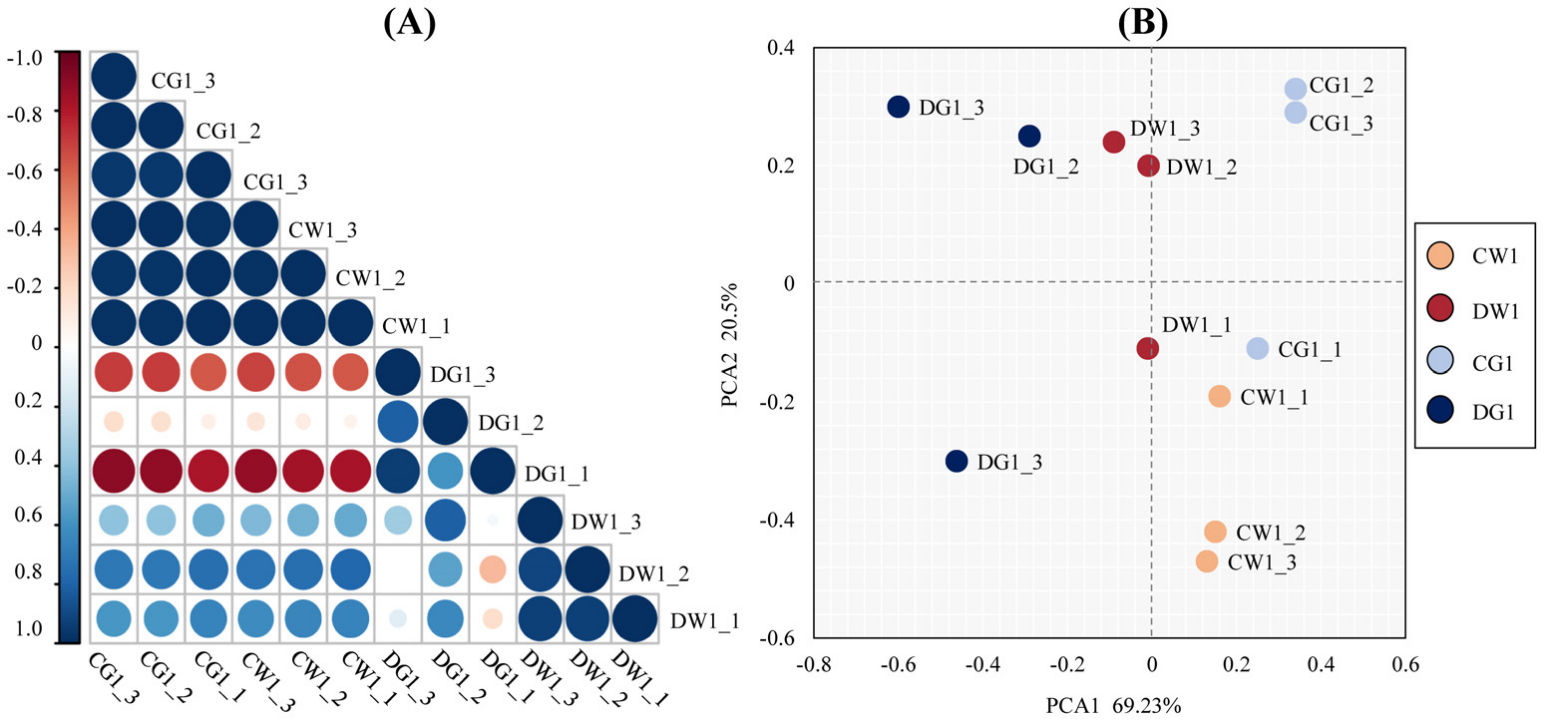
The repeatability and reliability test of the results of two cultivars and their replicates. **(A)** Pearson correlation coefficient analysis of biological replicates of two *C. pilosula* cultivars under different treatment conditions. The correlation coefficient (R^2^) between two corresponding (x- and y-axis) samples was calculated based on the TPM values of those samples. The x- and y- axes show the corresponding biological samples for different treatment conditions. **(B)** Principal component analysis (PCA) of the similarities and differences between the 12 samples used for RNA sequencing.

To test the repeatability and reliability of the results, we tested the relation of expression patterns among the drought treatment (D) and control (C) replicates of two cultivars by Pearson’s correlation test. The RNA-seq correlation coefficients of transcript per million (TPM), among DW1, DG1, CW1, and CG1 replicates, showed that the gene expression patterns were similar (**Figure 2A**), thus confirming the repeatability and reliability of evaluation results.

Furthermore, to analyze the similarities and differences between the 12 samples, principal component analysis (PCA) of all samples was performed (Mortazavi et al., 2008). The PCA results showed a clear separation between the two *C. pilosula* cultivars. Additionally, the replicates of each treatment clustered together (**Figure 2B**). These results showed that this experiment was reproducible and reliable.

### 3.3 Differential gene expression analysis of *C. pilosula* response to drought stress

The current study used TPM values ≥ 5 to determine the genes expressed. A total of 21,535 annotated gene transcripts were identified from the four treatments (DW1, DG1, CW1, and CG1). The Venn diagram (**Supplementary Figure S2**) shows the number of genes exclusively expressed in each treatment, overlapping genes between treatments, and common genes among all treatment combinations. Of these 21,535 gene transcripts, 63.71% (13720) were represented in all treatments. Before drought stress, 81.55% (17562) and 78.03% (16803) of the genes were expressed in W1 and G1, respectively. After drought stress, 80.85% (17412) and 79.81% (17188) were expressed in W1 and G1, respectively. A total of 735 genes were exclusively expressed in W1 after drought treatment (DW1), and a total of 1,437 genes were exclusively expressed in G1 after drought treatment (DG1). It is evident that a greater number of genes were expressed in variety G1 in response to drought stress, indicating that different mechanisms or pathways were activated to cope with the drought. Analyzing the differentially expressed genes in drought stress holds significant value.

The software R was used to explore differentially expressed genes (DEGs) between treatments at a standard fold change of less or equal to four (≤4) and false discovery rate (*P* < 0.05). Before drought treatment, we identified a total of 1,212 (809 upregulated and 403 downregulated) DEGs to be differentially expressed between the cultivar W1 and G1 (CW1_CG1). Under water-deficit conditions, 556 DEGs (241 upregulated and 315 downregulated) were observed between the cultivar G1 and W1 (DG1_DW1). From these results, we further compared the differences (in DEGs) between the cultivar G1 and W1. We identified 2,909 (1,369 upregulated and 1,540 downregulated) DEGs in the cultivar G1 (DG1_CG1).

**Figure 3:**
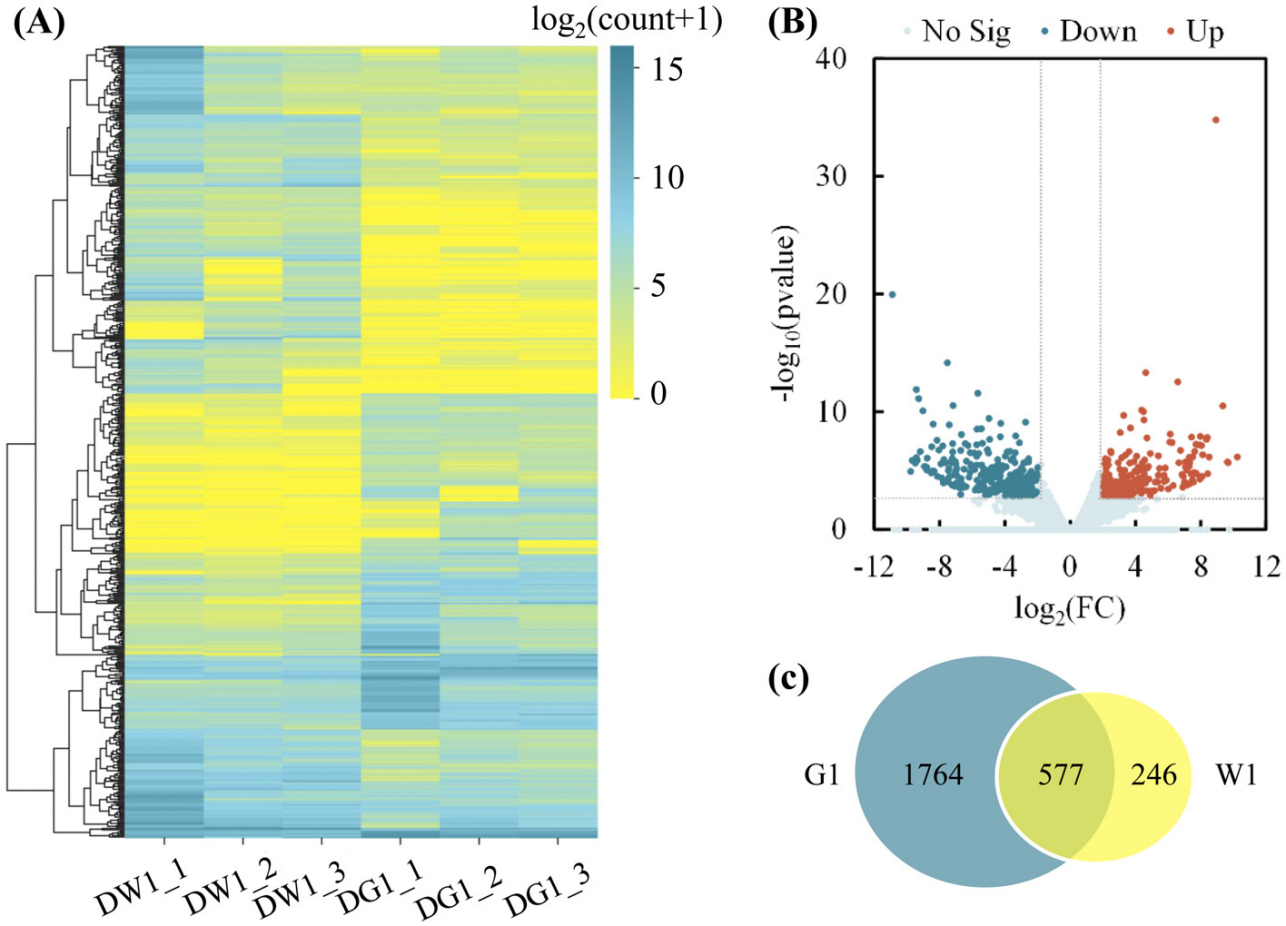
Clustering analysis of differentially expressed genes (DEGs) and number of DEGs in G1 and W1. **(A)** Clustering analysis of DEGs in two *C. pilosula* cultivars after drought stress treatment (DG1_DW1). The x-axis represents different samples. DW1_1, DW1_2, and DW1_3 refer to the three replicates of W1 drought-stressed; DG1_1, DG1_2, and DG1_3 refer to the three replicates of the G1 drought-stressed; and the y-axis represents the differential genes expressed. The scale bar indicates the DEG expression levels. The bluer the color, the higher the expression, whereas the yellower the color, the lower the expression. **(B)** Volcano plot showing the (log_2_FC, −log_10_FDR) expression of the DEGs in the DG1_DW1 experimental comparison. **(C)** Number of DEGs in drought-stressed W1 and G1 seedlings. The overlapping section of the Venn diagram shows the DEGs common to G1 and W1 seedlings after drought stress.

Clustering analysis of the DEGs of the DG1_DW1 experimental comparison showed that, after drought stress exposure, DEGs were grouped into 10 clusters, and more DEGs were downregulated than upregulated (**Figure 3A**). Additionally, analysis of the log_2_(FC) of these DEGs showed that the highest −log_10_ (Pvalue) was noted in the upregulated DEGs than downregulated DEGs (**Figure 3B**). Furthermore, a greater number of drought-responsive DEGs were observed in the G1 than in W1, and there were 1,764 specific DEGs, which were the specific drought-responsive DEGs of cultivar G1 (**Figure 3C**), and further research can be conducted on them.

### 3.4 KEGG analysis of DEGs related to *C*. *pilosula* response to drought stress

**Figure 4:**
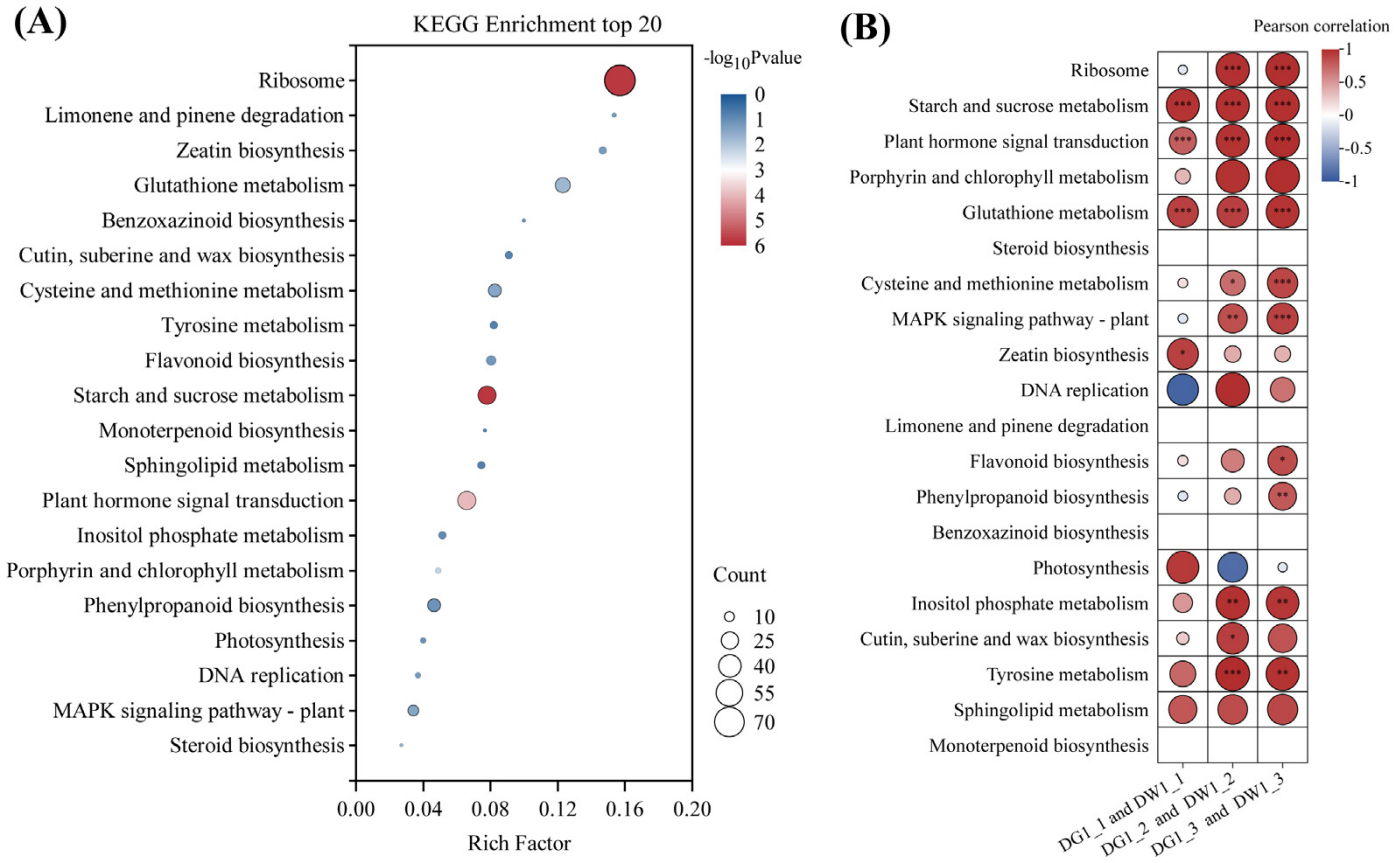
KEGG pathway enrichment and the Pearson correlation analysis of the DEGs of *C. pilosula* seedlings response to drought stress. **(A)** KEGG enrichment top 20 pathways of the DEGs. **(B)** Pearson correlation analysis of the DEGs. In the pathways of steroid biosynthesis, limonene and pinene degradation, benzoxazinoid biosynthesis, and monoterpenoid biosynthesis, fewer than three differentially expressed genes were enriched; hence, Pearson correlation analysis was not conducted. '*' indicates significance at the level of P < 0.05; '**' indicates significance at the level of P < 0.01; '***' indicates significance at the level of P < 0.001.

We conducted KEGG enrichment analysis on the 1,764 genes obtained above, and the results indicated that these genes were enriched in 84 KEGG pathways (**Supplementary Table S5**). The enrichment bubble plot (**Figure 4A**) for the top 20 metabolic pathways, as well as the heatmap (**Figure 4B**) showing the correlation between metabolic pathways and *C*. *pilosula* cultivars, revealed that the pathways related to plant stress resistance, such as starch and sucrose metabolism, plant hormone signal transduction, and glutathione metabolism pathway, were significantly positively correlated with the *C*. *pilosula* cultivars, indicating their involvement in the drought response of *C*. *pilosula*. Therefore, we conducted further analysis on the DEGs within these pathways.

**Figure 5:**
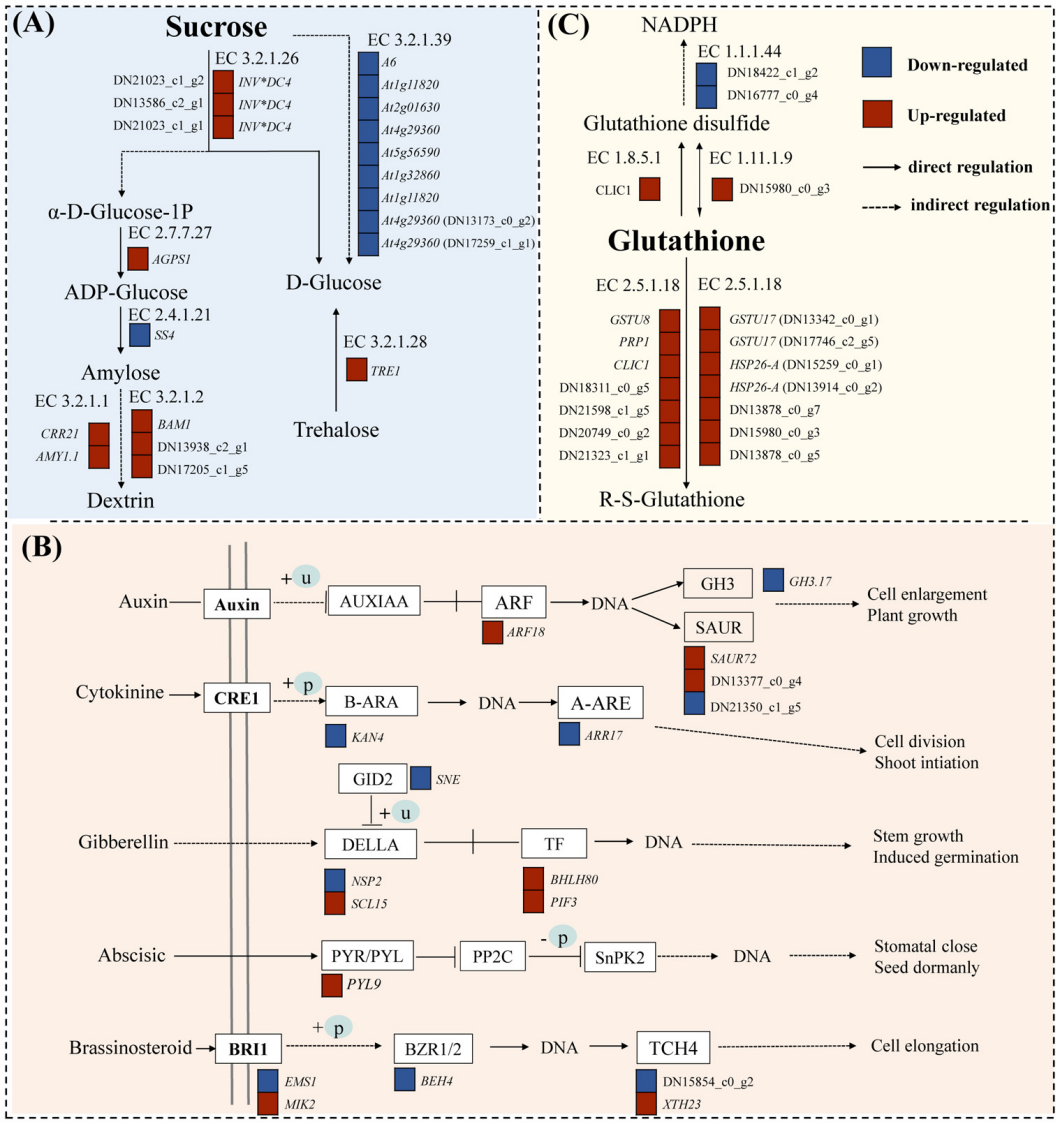
DEG pathway map of *C. pilosula* seedlings response to drought stress. **(A)** Starch and sucrose metabolism pathways. **(B)** Plant hormone signal transduction pathways. **(C)** Glutathione metabolism pathway.

Statistical analysis was conducted on the DEGs of starch and sucrose metabolism pathways (map00500) under drought stress (**Figure 5A**). The results showed that under drought stress, there were a total of 25 DEGs (10 upregulated and 15 downregulated). The up/downregulated genes included beta-amylase 1 (*BAM1*), pfkB-like carbohydrate kinase family, glucan endo-1,3-beta-glucosidase A6, hexokinase, and O- glycosyl hydrolases family. These genes are primarily involved in the metabolism of starch, sucrose, and cell walls in plants, making them important genes related to carbohydrate metabolism, which can rapidly respond under drought conditions, enhancing the energy metabolism of *C*. *pilosula* seedlings, thereby helping the plant resist drought stress.

A total of 25 DEGs (9 up- and 16 downregulated) were identified as involved in plant hormone signal transduction (map04075) (**Figure 5B**). The regulatory component of ABA receptor3-like gene (*PYL9*) was significantly upregulated after drought treatment in cultivar G1, and this gene has been confirmed to potentially participate in the response of plants to drought and other stresses in tomatoes (Infantes et al., 2022). And, the NSP-interacting kinase (SRF8) may be related to the plant signaling pathways, particularly involved in the plant’s response to environmental stresses (Li et al., 2019b). In addition, we found many transcription factors related to plant stress resistance and growth development regulation in this pathway, such as transcription factor KAN4 (*KAN4*), BES1/BZR1-like protein 4 (*BEH4*), ethylene-responsive transcription factor 1B (*ERF1B*), and transcription factor PIF3 isoform X1 (*PIF3*). Therefore, those above genes can be used as candidate genes for the response of cultivar G1 of *C*. *pilosula* seedlings to drought stress.

Statistical analysis was conducted on differentially expressed genes in the glutathione metabolism pathway (map00480) under drought stress (**Figure 5C**). The results showed that there were total of 17 DGEs (2 up- and 15 downregulated). A large number of glutathione S-transferase genes were found in this pathway, and these enzymes play an important role in the antioxidant and metabolic processes of plants, having been identified in multiple species (Kumar and Trivedi, 2018). Moreover, we found that GST_C domain-containing protein could be related to plant metabolism and stress resistance (Meng et al., 2023). These results indicated that the genes annotated in this pathway in our study were all involved in the resistance of cultivar G1 of *C*. *pilosula* seedlings to drought stress, helping the plant better adapt to environmental growth conditions.

### 3.5 The role of DEGs encoded transcription factors in drought resistance of *C*. *pilosula* seedlings

Research has shown that transcription factors play a key role in plant stress resistance (such as drought, salinity, and low temperature) primarily by regulating the expression of genes related to stress responses, thereby enhancing the plant’s adaptability. We analyzed the transcription factors (TFs) among 1,764 DEGs, and the results indicate that the TFs belonged to 5 families and mainly involved in *bHLH*, *ERF*, *MYB*, *PIF*, and *TCP* (**Table 1**). We found that most of these transcription factors play important roles in plant growth and responses, and they may be related to various physiological processes, thereby regulating the growth and development of *C*. *pilosula* seedlings drought tolerance.

**Table 1:** Transcription factors.

Gene_ID	Log_2_FC	KOEntry	Gene family	Name	Annotation
DN15527_c1_g3	−3.06	K14491	MYB	KAN4	Putative transcription factor KAN4
DN17717_c1_g4	−2.28	K14516	ERF	ERF1B	PREDICTED: ethylene-responsive transcription factor 1B
DN18631_c1_g4	2.29	K12126	bHLH	BHLH80	Transcription factor bHLH80 like
DN18739_c1_g5	2.03	K12126	PIF	PIF3	PREDICTED: transcription factor PIF3 isoform X1
DN15838_c1_g1	2.03	K16221	TCP	TCP9	Transcription factor TCP9-like
DN15518_c1_g1	−2.40	K16190	bHLH	FIT	Transcription factor FER-LIKE IRON DEFICIENCY-INDUCED transcription factor
DN16635_c0_g3	−3.77	K13434	ERF	CRF4	PREDICTED: ethylene-responsive transcription factor CRF2

The original ID is “TRINITY_DNXXXXX_X_X,” abbreviated as “DNXXXXX_X_X”.

### 3.6 qRT-PCR validation of DEGs

**Figure 6:**
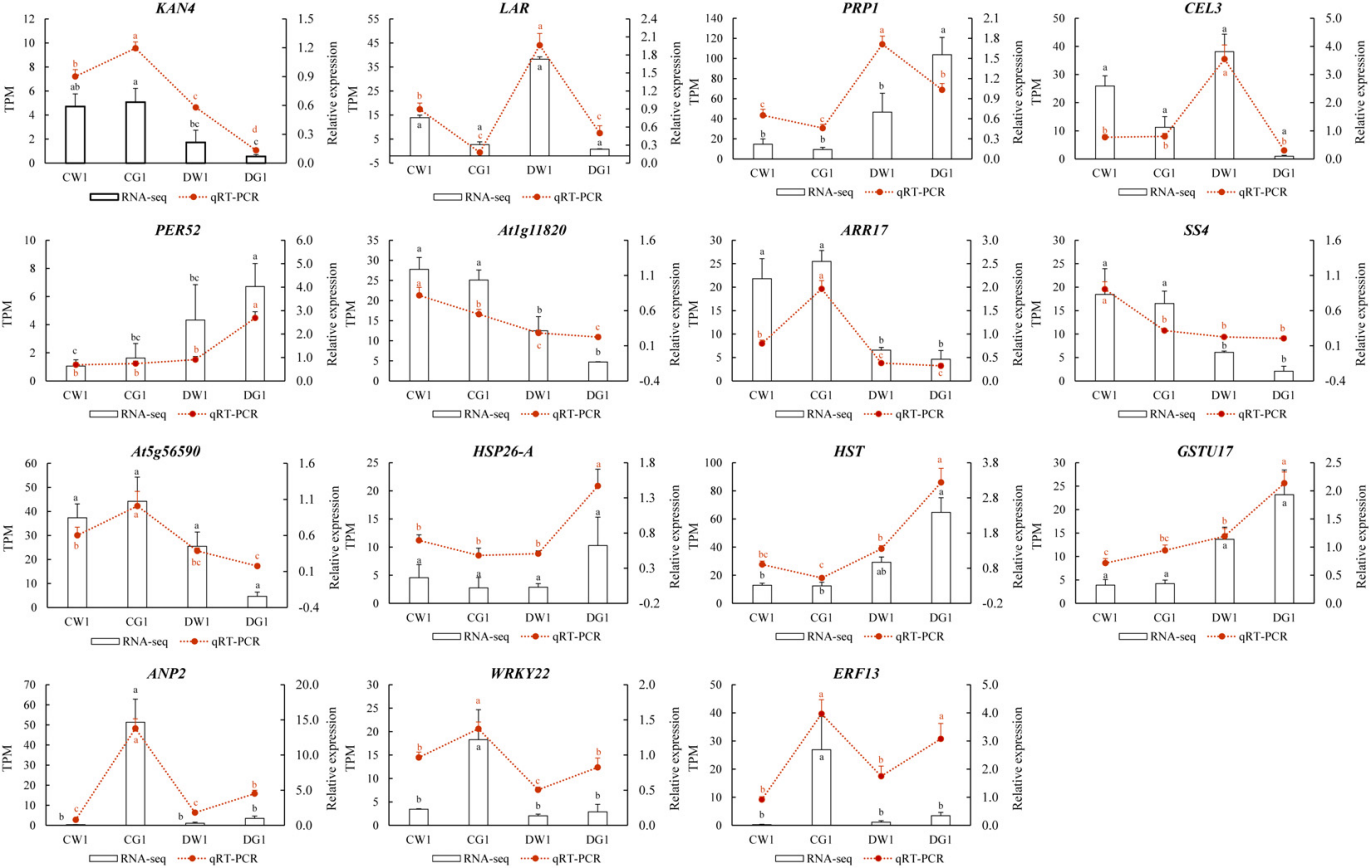
qRT-PCR validation of DEGs. Comparison of the relative expression level between RNA-seq and qRT-PCR in two cultivars under drought. The X-axis displays the selected genes, whereas the Y-axis shows the relative expression level.

To validate the transcriptome sequencing results, transcriptome data on 16 genes were randomly selected for qRT-PCR testing. The results indicated that the qRT-PCR expression changes of the 15 tested genes were consistent with the expression changes of the corresponding genes in the transcriptome sequencing data (**Figure 6**). Therefore, the transcriptome sequencing data in this study can be considered sufficiently reliable to serve as a foundation for differential expression analysis.

## 4 Discussion

Drought is one of the most damaging abiotic stressors for plants and triggered by insufficient rainfall, rising temperatures, and limited water availability. It is becoming an increasing problem due to global climate change. In response to drought stress, plants have evolved complex adaptive mechanisms at the physiological, biochemical, and molecular levels (Shan et al., 2013; Edmeades, 2013). However, the molecular mechanisms underpinning this phenomenon have remained elusive despite recent advances in molecular biology approaches (Bhanu et al., 2016). Here, we began with potting soil that had a moisture content exceeding 180 g/kg. By day 6, we reduced this moisture content to 130 g/kg or less, and by the end of the experiment, it was 123 g/kg or less. This process was designed to induce a water deficit in *C. pilosula* seedlings (**Figure 1**). Then, we determined the most sensitive period of *C. pilosula* to water deficiency through physiological index analysis and further studied the drought response patterns at the transcriptome level in two contrasting *C. pilosula* cultivars. We specifically identified the unique drought- responsive differentially expressed genes in the cultivar G1 seedlings. Furthermore, functional validation by qRT-PCR analysis corroborated the differential expression of these identified genes. Our findings provide deeper insights into the mechanisms of drought stress tolerance in *C. pilosula* seedlings, as well as providing a basis for further downstream analyses of the identified individual specific genes.

### 4.1 Clear divergence exists between *C*. *pilosula* cultivars G1 and W in their drought stress responses

Plants accumulate ROS under drought stress, which can impair chloroplast and mitochondrial functions, and subject the plant cells to oxidative damage, including lipid peroxidation, protein oxidation, and DNA damage. H_2_O_2_ is one of ROSs closely associated with oxidative stress. It is derived from disproportionation of superoxide anion, and the product of H_2_O_2_ has strong oxidation ability (Kissen et al., 2016; Yin et al., 2018). Throughout evolution, plants have developed an enzymatic antioxidant system to remove excess ROS, primarily involving ascorbate peroxidase (APX), SOD, POD, and CAT (Prerostova et al., 2020; Medina et al., 2021; Panchuk et al., 2002). Proline is an amino acid that accumulates in response to a variety of environmental factors, including water scarcity, salinity, low temperature, and heavy metal accumulation. Additionally, proline is a crucial variable amino acid in regulating the architectures of proteins and membranes, as well as scavenging reactive oxygen species (ROS) in drought-stressed organisms (Chun and Chandrasekaran, 2018). In *C. pilosula*, leaf osmotic adjustment substances (such as relative electrical conductivity, malondialdehyde, and automatic rate) showed a significant increase under drought days 1– 6. Meanwhile, the proline, APX, SOD, POD, and CAT activities increased on days 1 –4, helping to maintain ROS balance during the early stage of drought stress. However, their activities decreased after 4 days, indicating that the protective enzyme system is highly time dependent in response to drought stress in *C. pilosula* seedlings. Moreover, the cultivar G1 exhibited relatively higher protective enzyme activity and the enhanced ROS quenching competency of its cells resulted in greater cell membrane integrity and relatively lower levels of osmotic adjustment substances in leaves. Consequently, cultivar G1 seedlings with better drought stress endurance than cultivar W1.

Chlorophyll a is a key pigment that is involved in multiple chlorophyll–protein complexes within plants’ photochemical and carbon fixation systems (Bano et al., 2013). It is instrumental in capturing light energy during photosynthesis. Chlorophyll b is primarily used in the creation of light-harvesting chlorophyll–protein complexes within the photochemical system. These complexes assist in absorbing light energy and transferring it to chlorophyll a, thereby facilitating the photosynthetic process. Despite variations based on plant characteristics and conditions, chlorophyll content serves as an effective indicator of desiccation tolerance. A common trait under drought stress conditions is a decrease in leaf chlorophyll content. This reduction in chlorophyll levels is often linked to oxidative stress and chlorophyll damage. The chlorophyll content is closely linked to photosynthetic activity, and alterations in chlorophyll levels can significantly affect a plant’s overall photosynthetic performance. There was a difference in chlorophyll content between the two *C. pilosula* cultivars under drought treatment. The chlorophyll a/b ratio in cultivar G1 increased, whereas the a/b ratio remained unchanged in cultivar W1. The increase in the a/b ratio of cultivar G1 is attributed to a slight increase in chlorophyll a, which is anticipated to enhance the activity of carbon fixation systems. This suggests that the drought stress conditions were not severe enough to significantly reduce photosynthesis of cultivar G1.

### 4.2 Transcriptomic analysis of the molecular mechanisms of drought resistance in *C. pilosula* seedlings

Stress perception is the first step to ensure plant survival to abiotic stress exposure (Frolov et al., 2017). The stress is firstly perceived by the receptors located on cell membranes, such as for GPCRs, PLKs, HKs, ABA, and CAS. The signals are then transduced downstream, leading to the generation of secondary messengers including K^+^, Ca^2+^, sugars, ROS, cyclic nucleotides, and inositol phosphates (Wu et al., 2017). The secondary messengers trigger the corresponding signaling pathways to transduce the signals (Chaves et al., 2009). Central to the signal transduction machinery are protein kinases and phosphatases that mediate protein phosphorylation and dephosphorylation, respectively. The calcium-dependent protein kinases (CDPKs), abscisic acid-activated signaling (ABA), and mitogen- activated protein kinase (MAPK) pathways are vitally involved in plant abiotic stress responses (Jonak et al., 1996; Dudhate et al., 2018).

In this study, a total of 25 DEGs related to plant hormone signal transduction were identified only in cultivar G1 seedings under drought (**Figure 5B**). We found that the regulatory component of ABA receptor3-like gene (*PYL9*) was significantly upregulated, NSP-interacting kinase (*SRF8*) related to the plant signaling pathways was significantly downregulated, and two TFs (*KAN4* and *ERF1B*) were significantly downregulated, whereas two TFs (*BHLH80* and *PIF3*) were significantly upregulated. When stress occurs, a large amount of ABA is produced in the cell, and PYL protein, as a receptor for ABA, first senses and binds to it. Then, the PYL–ABA complex reacts with clade A protein phosphatase 2C (*PP2C*). The binding of PP2C to form the PYL-ABA-PP2C ternary complex results in the loss of activity of PP2C, thereby relieving the inhibitory effect on the kinase sucrose non-financial 1- related protein kinase subfamily 2 (*SnRK2s*) and activating the kinase *SnRK2s* (Schweighofer et al., 2004; Melcher et al., 2009). The activated *SnRK2s* phosphorylate downstream transcription factors or effector proteins, thereby initiating the expression of ABA responsive genes (Fujita et al., 2012), inducing stomatal closure and reducing transpiration, thereby improving plant stress resistance.

Starch and sucrose are important forms of carbohydrate storage in plants, and plants can utilize carbohydrate reserves reasonably by regulating metabolic pathways to maintain the energy and material supply required for growth and development. In drought-resistant varieties, the expression of sucrose metabolism-related genes is upregulated under drought stress, which helps to scavenge ROS and alleviate oxidative damage (Thomas and Beena, 2021). Genes related to starch and sucrose metabolism pathways are significantly enriched under drought stress (Cao et al., 2022). Liang et al. (2021) suggests that carbohydrates served as biomarkers for drought stress response and enhanced drought tolerance in *C*. *pilosula*. In this study, there were a total of 25 DEGs in G1 cultivar seedings enrichment in starch and sucrose metabolism pathways, suggesting that G1 cultivar seedings can maintain growth requirements under drought stress through more efficient energy metabolism.

In addition, we have known that glutathione (GSH) is an important antioxidant that can scavenge reactive oxygen species (ROS) and free radicals, preventing these substances from causing damage to cellular components (Gaucher et al., 2018). Under abiotic stresses such as high salinity, drought, and low temperatures, the levels of reactive oxygen species in plants increase, leading to cellular damage (Hasanuzzaman et al., 2020). Additionally, the activity of antioxidant enzymes such as glutathione S-transferases (GST) increases, helping plants eliminate excess reactive oxygen species and mitigate oxidative damage (Khan et al., 2022). Glutathione plays a crucial role in the ascorbate (AsA) and glutathione cycle, participating in the reduction of H_2_O_2_ and further enhancing the plant’s antioxidant defense mechanisms (Valero et al., 2016). Therefore, glutathione not only is an important antioxidant in plants but also plays a key role in responding to various environmental stresses, helping plants maintain physiological balance and growth. Our results suggested that 17 DEGs were identified as involved in glutathione metabolism pathway, and 9 glutathione S-transferase genes were significantly upregulated in cultivar G1 of *C*. *pilosula*. Transcriptome analysis under drought stress revealed that drought-resistant bananas exhibited significantly upregulated expression of glutathione metabolism-related genes compared with sensitive varieties (Muthusamy et al., 2016). Furthermore, comparable findings have been reported in a large number of plants species, such as Arabidopsis (Meng et al., 2023), chickpea (Singh et al., 2023), rice (Wang et al., 2022), and maize (Anjum et al., 2017).

### 4.3 Transcription factor- related genes are a critical component of drought response machinery

The drought stress response is controlled by complex regulatory networks in plants. Transcription factors (TFs) are important regulators in this network, playing a pivotal role by activating or inhibiting the expression of downstream stress-related target genes (Wang et al., 2016). To date, numerous TFs have been reported being involved in the regulation of drought stress tolerance in plants, including MYB, ERF, and bHLH. The BHLH family is the largest transcription factor family in eukaryotes (Sun et al., 2018b) and plays an important role in regulating plant drought resistance. For example, recent studies have shown that the peanut bHLH transcription factor *AhbHLH112* can enhance peanut drought resistance, and drought can significantly induce its expression (Li et al., 2021). Moreover, the maize bHLH transcription factor *ZmPTF1* regulates maize tolerance to drought stress by promoting root development and ABA synthesis (Li et al., 2019a). Research in *Arabidopsis* and some crops has shown that MYB transcription factors are involved in response to drought stress, such as regulating stomatal movement, leaf development, flavonoid, and cell wall synthesis (Baldoni et al., 2015). Chen et al. (2022) suggest that the MYB family transcription factor *SlMYB55* is an ABA and drought- responsive gene, and silencing the expression of *SlMYB55* can significantly enhance tomato drought resistance.

Numerous studies have shown that ERF transcription factors play an important role in plant stress response. For instance, the rice *OsERF922* negatively regulates plant salt tolerance by disrupting Na^+^/K^+^ homeostasis and mediating the ABA signaling pathway (Liu et al., 2012). In our study, we identified a total of seven drought- related transcription factors in the *C*. *pilosula*, belonging to five gene families, namely, bHLH, ERF, MYB, PIF, and TCP families. Furthermore, one DEG related to bHLH, PIF, and TCP was upregulated in cultivar G1, whereas two DEGs related to ERF and one DEG related to bHLH and MYB were downregulated. These studies indicated that those specific TFs may play essential roles in plant response to environmental stress. Therefore, the different expression of these specific TFs only in cultivar G1 may be one of the pivotal reasons for its greater drought tolerance compared with cultivar W1.

### 4.4 Proposed molecular model of *C*. *pilosula* seedling drought stress tolerance

**Figure 7:**
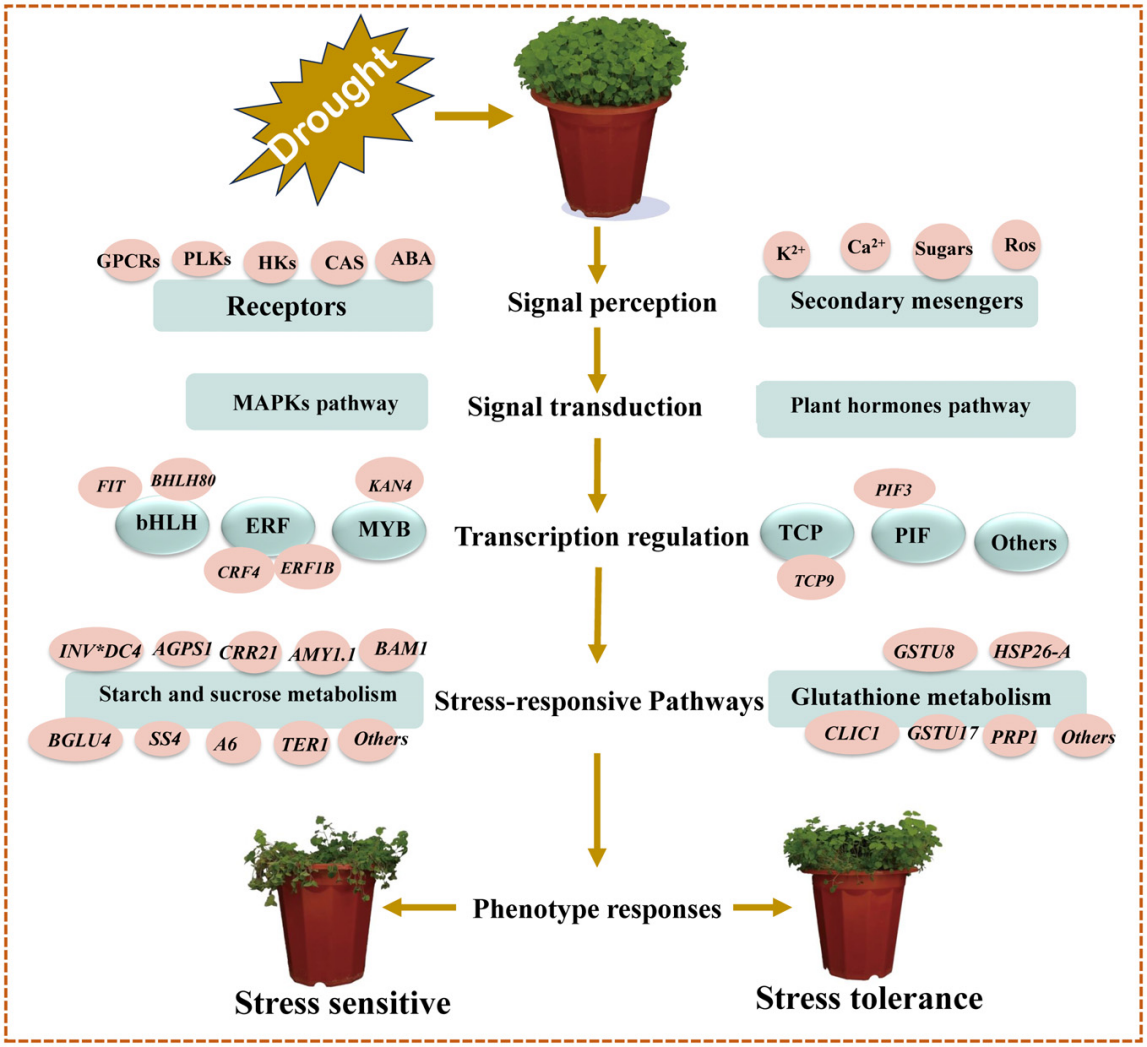
Schematic molecular model of *C*. *pilosula* seedlings drought stress tolerance. This model was developed based on our key putative components of drought response identified in this study, supported by previously described schemes of plant abiotic stress response pathways/networks (Wang et al., 2016; Shinde et al., 2018).

Based on our main findings of the key drought- responsive DEGs and their associated pathways/networks, and the relevant published citations contained in this study, we have developed a molecular model for drought stress tolerance in *C*. *pilosula* seedlings as shown in **Figure 7**.

## 5 Conclusions

In this study, physiological and comparative transcriptome analyses were performed on 4-week seedling leaf tissues of *Codonopsis pilosula* cultivars ‘G1’ and ‘W1’ seedings in drought treatment for 4 days to explore the key genes responsible for the differences in response to drought between these two cultivars. Resultantly, cultivar G1 seedlings maintained comparatively higher proline contents, chlorophyll content, and greatly increased peroxidase activity but decreased malondialdehyde content and leaf relative electrolyte conductivity, than cultivar W1 seedlings. Using an RNA sequencing (RNA-seq)-based approach, we identified a total of 21,535 genes that were expressed and 1,764 differentially expressed genes (DEGs) only in G1 under drought stress. All those DEGs got annotation and functional classification based on different databases. We suggest that drought-induced changes in osmoregulation to prevent water deficit and enhance the defense capacity of the antioxidant system are the strategies of *C*. *pilosula* cultivar G1 in response to drought stress. We found hormonal regulation (ABA); regulation of transcription factors, such as *KAN4*, *BHLH80*, and *ERF1B*; and regulation of drought-responsive genes, such as glutathione metabolism- and starch and sucrose metabolism- related genes. The functions of these genes and the physiological responses of plants provide a basis for preliminarily explaining the mechanism of physiological changes in *C*. *pilosula* under drought stress and breed new *C*. *pilosula* cultivars.

## Data Availability

The datasets presented in this study can be found in online repositories. The names of the repository/repositories and accession number(s) can be found in the article/**Supplementary Material**.
