# Supplementary material for: Codonopsis pilosula seedling drought- responsive key genes and pathways revealed by comparative transcriptome
Source: Front Plant Sci. 2024 Oct 30;15:1454569. doi: 10.3389/fpls.2024.1454569 (PMC11561192; doi:10.3389/fpls.2024.1454569)
Supplement: Supplementary file 2 [file Table2.docx]

*Codonopsis pilosula* Seedlings Drought-Responsive Key Genes and Pathways Revealed by Comparative Transcriptome

Hongyan Wang^1^, Yuan Chen^1*^, Lanan Liu^1,2^, Fengxia Guo^1*^, Wei Liang^1^, Linlin Dong^3^, Pengbin Dong^1^, Jiali Cheng^1^

*Correspondence:

Yuan Chen, Fengxia Guo

Agronomy College

Gansu Agricultural University

Lanzhou 730070, Gansu, China.

Emails: [chenyuan@gsau.edu.cn](mailto:chenyuan@gsau.edu.cn), [guofx@gsau.edu.cn](mailto:guofx@gsau.edu.cn);

**Figure S1** **The climatic conditions throughout the entire experimental period**

**Figure S2 Profile of gene expression by two *C. pilosula* cultivars drought treatment**


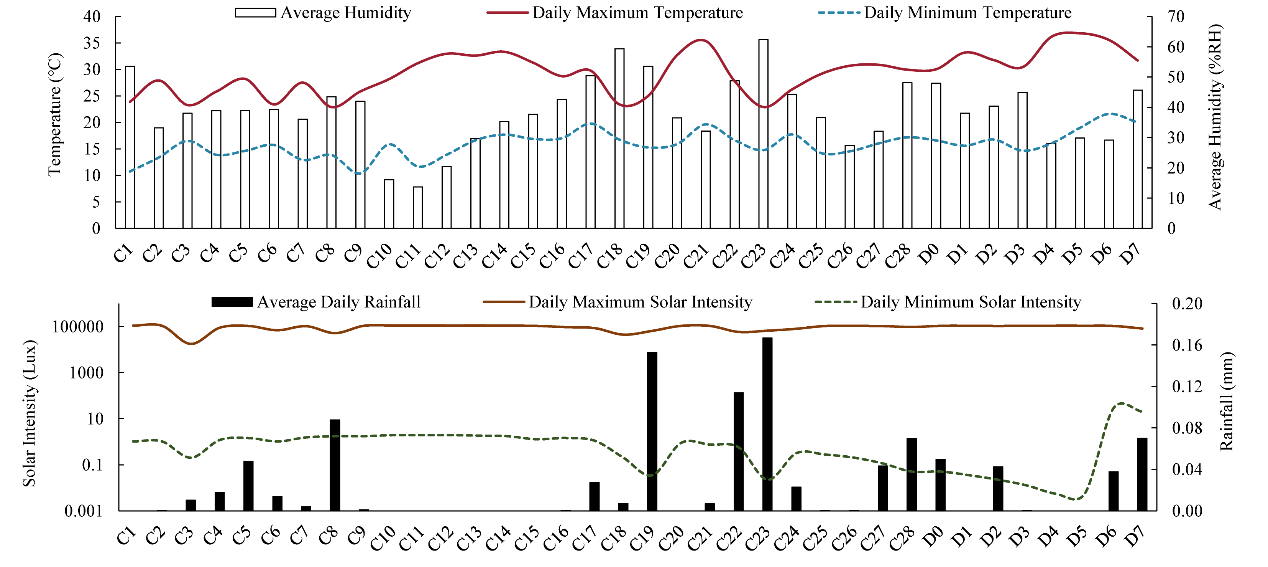


**Figure S1** **The climatic conditions throughout the entire experimental period**

C1~C28 refer to the growth period of *Codonopsis* seedlings, D1~D7 refer to the drought stress period of *Codonopsis* seedlings.


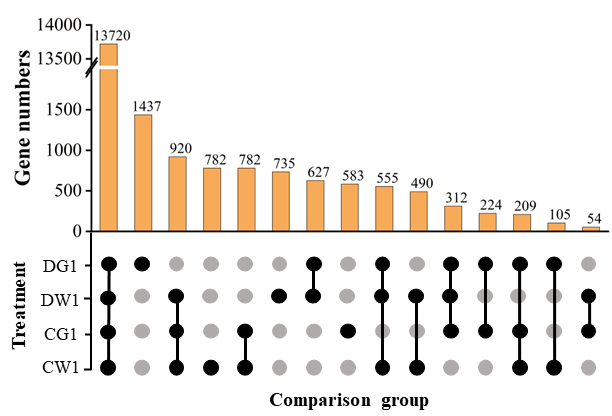


**Figure S2** **Profile of gene expression by two *C. pilosula* cultivars drought treatment**

The gene expression profile is illustrated as the number of transcriptomic responses using a Venn diagram. A total of 21535 genes were expressed. Drought treatments are labelled Control (C) and Drought (D). The two *C. pilosula* cultivars are labelled “W1” and “G1”, respectively. The biological samples of four combinations are CW1, CG1, DW1, and DG1, respectively.
